# Laparoscopic vs. open distal gastrectomy for locally advanced gastric cancer: A systematic review and meta-analysis of randomized controlled trials

**DOI:** 10.3389/fsurg.2023.1127854

**Published:** 2023-02-17

**Authors:** Yong Yan, Caiwen Ou, Shunwang Cao, Yinggang Hua, Yanhua Sha

**Affiliations:** ^1^ Department of General Surgery, Guangzhou Red Cross Hospital, Jinan University, Guangzhou, China; ^2^ Department of Laboratory Medicine, The Second Affiliated Hospital of Guangzhou University of Chinese Medicine, Guangzhou, China

**Keywords:** laparoscopic distal gastrectomy, open distal gastrectomy, advanced gastric cancer, meta-analysis, systematic review, stomach neoplasms, laparoscopy

## Abstract

**Objective:**

The aim of this systematic review and meta-analysis is to compare the short- and long-term outcomes of laparoscopic distal gastrectomy (LDG) with those of open distal gastrectomy (ODG) for patients with advanced gastric cancer (AGC) who exclusively underwent distal gastrectomy and D2 lymphadenectomy in randomized controlled trials (RCTs).

**Background:**

Data in published meta-analyses that included different gastrectomy types and mixed tumor stages prevented an accurate comparison between LDG and ODG. Recently, several RCTs that compared LDG with ODG included AGC patients specifically for distal gastrectomy, with D2 lymphadenectomy being reported and updated with the long-term outcomes.

**Methods:**

PubMed, Embase, and Cochrane databases were searched to identify RCTs for comparing LDG with ODG for advanced distal gastric cancer. Short-term surgical outcomes and mortality, morbidity, and long-term survival were compared. The Cochrane tool and GRADE approach were used for evaluating the quality of evidence (Prospero registration ID: CRD42022301155).

**Results:**

Five RCTs consisting of a total of 2,746 patients were included. Meta-analyses showed no significant differences in terms of intraoperative complications, overall morbidity, severe postoperative complications, R0 resection, D2 lymphadenectomy, recurrence, 3-year disease-free survival, intraoperative blood transfusion, time to first liquid diet, time to first ambulation, distal margin, reoperation, mortality, or readmission between LDG and ODG. Operative times were significantly longer for LDG [weighted mean difference (WMD) 49.2 min, *p* < 0.05], whereas harvested lymph nodes, intraoperative blood loss, postoperative hospital stay, time to first flatus, and proximal margin were lower for LDG (WMD −1.3, *p* < 0.05; WMD −33.6 mL, *p* < 0.05; WMD −0.7 day, *p* < 0.05; WMD −0.2 day, *p* < 0.05; WMD −0.4 mm, *p* < 0.05). Intra-abdominal fluid collection and bleeding were found to be less after LDG. Certainty of evidence ranged from moderate to very low.

**Conclusions:**

Data from five RCTs suggest that LDG with D2 lymphadenectomy for AGC has similar short-term surgical outcomes and long-term survival to ODG when performed by experienced surgeons in hospitals contending with high patient volumes. It can be concluded that RCTs should highlight the potential advantages of LDG for AGC.

**Systematic Review Registration:**

PROSPERO, registration number CRD42022301155.

## 1. Introduction

In the year 1994, Kitano et al. described the first laparoscopic distal gastrectomy (LDG) for a patient with early gastric cancer (EGC) in Japan (1). Since some randomized controlled trials (RCTs) reported feasible short-term outcomes and similar long-term survival rates for both LDG and open distal gastrectomy (ODG), LDG became a standard technique for the treatment of EGC (2–4). In hospitals with high patient volumes, surgeons with an extensive experience of laparoscopic procedures have performed LDG for locally advanced gastric cancer (AGC). Although some studies have demonstrated the safety of LDG for AGC, short-term surgical outcomes and long-term survival rates of LDG vs. ODG are still inconclusive (5–7). Several previous meta-analyses have compared the surgical and survival outcomes of LDG vs. ODG for AGC. However, these studies included both RCTs and nonrandomized comparative studies, as well as the combined data of both early and advanced cases. Furthermore, some of the meta-analyses included studies that reported the combined outcomes of the different extents of resection, such as total and proximal as well as distal gastrectomy cases (8–10). Thus, these meta-analyses were subject to a high amount of bias for evaluating the oncologic safety and efficacy specific to LDG and advanced distal gastric cancer. Furthermore, the shortage of long-term survival outcomes in these studies prevented making out a case for obtaining complete support for LDG as a feasible procedure. Recently, several RCTs that compared LDG with ODG included AGC patients specifically planned for distal gastrectomy, with D2 lymphadenectomy being reported and updated with long-term outcomes (11–13). Therefore, the aim of this systematic review and meta-analysis is to include RCTs for the purpose of comparing the short- and long-term outcomes of LDG with those of ODG with D2 lymphadenectomy in adults diagnosed with advanced distal gastric cancer.

## 2. Methods

This meta-analysis followed the Cochrane Handbook for Systematic Review and Meta-Analysis as well as Preferred Reported Items for Systematic Reviews and Meta-analysis (PRISMA) recommendations (14, 15). This study was registered at PROSPERO (CRD42022301155).

### 2.1. Search strategy

We searched the English-language literature published up to December 2021 in PubMed, Embase, and Cochrane with the following terms: [laparoscopic OR laparoscopy OR laparoscopically assisted OR minimal invasive surgery] AND [open OR conventional OR open conventional surgery] AND [gastrectomy OR distal gastrectomy OR stomach resection] AND [gastric cancer OR stomach cancer OR gastric carcinoma OR stomach carcinoma OR advanced gastric cancer OR locally advanced gastric cancer] AND [randomized controlled trial OR randomized clinical trial].

### 2.2. Eligibility criteria

RCTs that evaluated open to laparoscopic/laparoscopy-assisted distal gastrectomy with D2 lymphadenectomy for locally AGC were included. Only adult patients were included. The AGC definition used was histologically proven gastric cancer, no distant metastasis, and pretherapeutic stage equal to or greater than 2 (American Joint Committee on Cancer/Union for International Cancer Control stage). Studies including total and proximal as well as distal resections were excluded, unless they provided separate outcomes for patients who underwent distal gastrectomy. Studies that included those who underwent gastrectomy for EGC, gastrointestinal stroma tumors, neuroendocrine tumors, or benign lesions were excluded.

### 2.3. Data extraction

The primary outcomes were intraoperative complications, overall postoperative complications, severe postoperative complications, oncologic outcomes (such as lymph node retrieval, D2 lymphadenectomy, R0 resection, and recurrence rate), and long-term survival. A specific complication was diagnosed on the basis of image-based evaluation or obvious clinical evidence according to the included trials reported. Furthermore, secondary outcomes comprised operative time, intraoperative blood loss, intraoperative blood transfusion, postoperative hospital stay, time to first flatus, time to first liquid diet, time to first ambulation, surgical margin, reoperation, mortality, readmission, and type of postoperative complications. Parameters such as study characteristics, demographic characteristics, inclusion and exclusion criteria, surgical details, surgeons' experience, and surgical quality control were determined by using a standardized data extraction sheet.

### 2.4. Quality assessment

The Cochrane risk-of-bias tool was employed for assessing the quality of the methodology used. The Grading of Recommendations Assessment, Development, and Evaluation (GRADE) approach was used for assessing the quality of evidence. The outcomes were assessed in terms of risk of bias, inconsistency, indirectness, imprecision, and publication bias. In case of serious bias, evidence quality was downgraded. All discrepancies were resolved by way of discussion and consensus.

### 2.5. Statistical analysis

The software Review Manager version 5.4 was used to analyze the data. For dichotomous data, the odds ratio (OR) with 95% confidence interval (CI) was calculated. For continuous data, the weighted mean difference (WMD) with 95% CI was calculated. The hazard ratio (HR) was used by implementing a generic inverse variance method to analyze survival outcomes. Heterogeneity was assessed by employing *I*^2^, with values of more than 50% indicating significant heterogeneity. The random effects model was used when *I*^2^ was more than 50%, and the fixed effects model was used when *I*^2^ was less than 50%. A value of *p *< 0.05 was considered statistically significant. Sensitivity analysis and estimation of publication bias were also performed.

## 3. Results

### 3.1. Study selection

**Figure 1:**
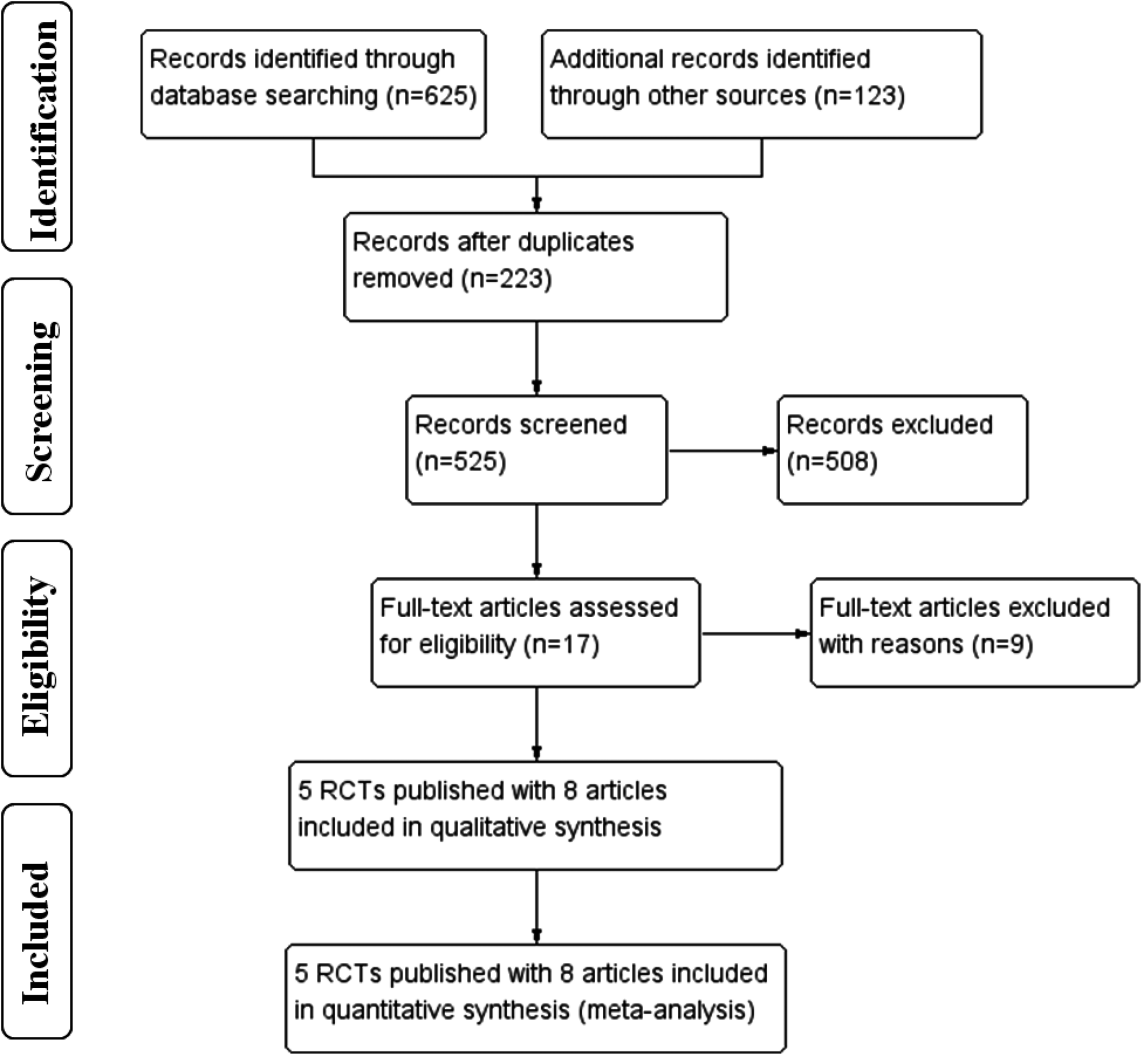
Selection flow diagram.

The selection flow diagram is shown in Figure 1. A total of 748 articles were retrieved, 17 articles were full-text reviewed, and 9 were excluded on grounds of ineligibility. After study selection, five eligible RCTs (16–20) published with eight full-length articles met the inclusion criteria and these were finally included, as the RCT of CLASS-01 had additional 3-year (11) and 5-year (12) outcomes published and the RCT of KLASS-02 had additional 3-year (13) outcomes published.

### 3.2. Study characteristics

The five two-armed RCTs included a total of 2,746 patients and were conducted in China (3) and Korea (2). The demographic characteristics are summarized in Table 1. No significant differences in baseline characteristics were found, except in the trial reported by Li et al., in which it was found that the LDG group contained more patients with advanced-stage disease and who were assessed by using the clinical TNM staging system. However, ypT, ypN, and ypTNM stages were similar between the two groups. The mean follow-up time was 71 months in the CLASS-01 trial (12), 36 months in the KLASS-02 trial (13), and 38 months in the trial reported by Park et al. (18).

**Table 1:** Demographic and baseline characteristics of patients in included studies.

Study	Site	Intervention	No. (M/F)	*p*-value	Age (years)	*p*-value	BMI (kg/m^2^)	*p*-value	ASA score (I/II/III)	*p*-value	Tumor size (cm)	*p*-value	T stage (T1/T2/T3/T4)	*p*-value	N stage (N0/N+)	*p*-value	TNM stage (I/II/III/IV)	*p*-value
CLASS-01	China multicenter	LDG	519 (380/139)	0.019	56.5 ± 10.4	0.306	22.7 ± 3.2	0.940	—	—	4.0 ± 2.0	0.580	—–	—	—	—	151/77/219/11	0.548
		ODG	520 (346/174)		55.8 ± 11.1		22.7 ± 3.2		—		4.0 ± 2.1		—		—		152/138/221/8	
KLASS-02	Korea multicenter	LDG	492 (351/141)	0.574	59.8 ± 11.0	0.620	23.5 ± 2.9	0.203	239/228/25	0.933	4.6 ± 2.5	0.779	137/104/132/119	0.731	223/269	0.999	178/148/166/0	0.310
		ODG	482 (335/147)		59.4 ± 11.5		23.7 ± 3.3		235/225/22		4.6 ± 2.3		125/113/135/109		219/263		165/167/150/0	
Wang et al. (2019)	China multicenter	LDG	222 (144/78)	0.338	59.4 ± 12.4	0.291	23.1 ± 3.1	0.243	96/124/2	0.209	3.6 ± 1.8	0.106	58/45/65/54	0.656	100/122	0.662	75/63/80/4	0.982
		ODG	220 (133/87)		60.6 ± 10.2		23.5 ± 3.3		83/131/6		3.9 ± 2.2		52/35/71/62		93/127		68/63/83/6	
Park et al. (2018)	Korea multicenter	LDG	100 (69/31)	0.846	58.6 ± 8.9	0.648	23.7 ± 3.0	0.419	—	—	—	—	—	—	—	—	42/29/28/1	0.553
		ODG	96 (65/31)		60.1 ± 8.2		23.3 ± 3.1		—		—		—		—		36/33/23/4	
Li et al. (2019)	China single-center	LDG	47 (33/14)	0.740	59.0 ± 3.2	0.440	23.5 ± 1.0	0.670	—	—	2.5 ± 0.4	0.500	8/9/17/9	0.97	24/23	0.07	13/18/12/0	0.64
		ODG	48 (33/15)		61.0 ± 2.2		22.6 ± 0.9		—		2.5 ± 0.5		10/7/19/9		32/16		16/19/10/0	

BMI, body mass index; LDG, laparoscopic distal gastrectomy; ODG, open distal gastrectomy; No. (M/F), number of patients (male/female).

The recruitment and surgical details are summarized in Supplementary Table S1. Four of the included trials were multicenter studies (16–19), whereas the trial reported by Li et al. (20) was a monocentric study. All included patients suffered from histologically proven primary gastric adenocarcinoma and had clinical tumor stages cT2 to cT4a and cM0. All trials included patients with clinical lymph node staging cN0 to cN3, except the KLASS-02 trial (17), which only included patients with cN0 and cN1. Patients with a history of major upper abdominal surgery or previous gastric resection, an American Society of Anesthesiologists (ASA) score of more than 3, and younger than 18 years of age were excluded. Patients aged more than 80 years were excluded in the CLASS-01 (16) and KLASS-02 trials (17) and in those reported by Park et al. (18) and Li et al. (20), whereas Wang et al. (19) did not exclude those above 80 years from participating in the study. All RCTs only included patients for whom distal gastrectomy with D2 lymphadenectomy was planned. The CLASS-01, KLASS-02, Park et al., and Wang et al. trials did not include patients who received preoperative chemotherapy or radiochemotherapy. In the trial reported by Li et al., both LDG and ODG for AGC patients after neoadjuvant chemotherapy were compared and assessed (20). All RCTs reported that adjuvant chemotherapy was administered to patients when there were no contraindications, whereas data on the number of patients who actually received chemotherapy were not available. Surgical details were reported in all studies, in which standard distal gastrectomy with D2 lymph node dissection was based on the Japanese gastric cancer treatment guidelines, and the reconstruction method was selected from the standard procedures of Billroth I/II or Roux-en-Y depending on the surgeon's preference. Extracorporeal anastomosis using a procedure called minilaparotomy was recommended during the performance of LDG in the CLASS-01 trial (16), whereas there was the option of selecting the extracorporeal or intracorporeal method for anastomosis according to the surgeon's discretion in KLASS-02 (17).

### 3.3. Study quality

**Figure 2:**
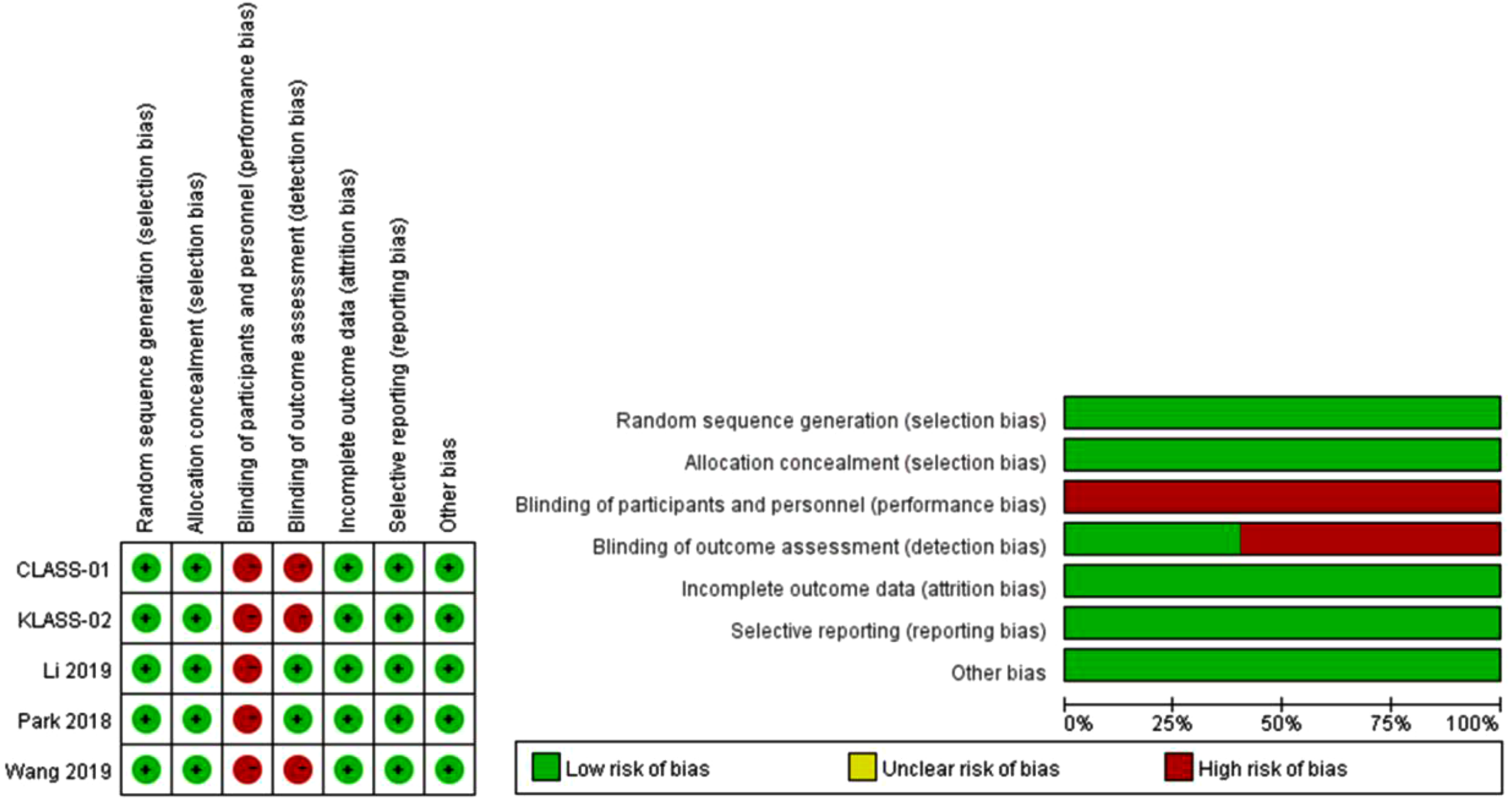
Risk of bias assessment.

The risk of bias assessment is presented in Figure 2. Random sequence generation and allocation concealment were adequate in all RCTs. All included RCTs had a high risk of bias for blinding, except in RCTs reported by Park et al. and Li et al. A blinded assessment of the primary outcome (noncompliance rate of lymph node dissection) was provided by blinded observers in the trial reported by Park et al. (18), and the outcome assessment by pathologists and radiologists were blinded in the Li et al. trial (20). Thus, within the Park et al. and Li et al. trials, the risk for performance bias was high and that for detection bias was low. All included RCTs had a low risk for attrition and reporting bias.

### 3.4. Surgeons' qualification and quality control

Surgeons' qualification and control measures of surgical quality are summarized in Supplementary Table S2. All included studies reported both the surgeons' experience level for participating in their respective studies and the measures for controlling the quality of D2 lymphadenectomy. Video recordings and photographs of the surgical site were preserved for assessing surgical quality. Through reviewing the video records, a list of checkpoints was used for evaluating completeness of D2 lymphadenectomy in the trial of Park et al. (18). It was found that surgeons' experience in LDG at the start of the trials differed among studies. Within the single-center RCT reported by Li et al., one surgeon performed all surgeries and had previous experience of performing more than 600 laparoscopic gastrectomies (20). Surgeons had performed more than 50 LDGs and ODGs in the CLASS-01, KLASS-02, and Wang et al. RCTs, whereas the Park et al. RCT required surgical experience for performing only 30 laparoscopic gastrectomies. In the CLASS-01 trial, surgeons were qualified by assessing unedited videos, and only those institutions with an annual surgical volume of at least 300 gastrectomies for AGC participated in the trial. In the KLASS-02 trial, surgeons were qualified by conducting a clinical trial (KLASS-02-QC, NCT01283893) (21), and an annual surgical volume of more than 80 cases was the requirement for participation.

### 3.5. Open conversion

All included RCTs reported open conversion rates for the LDG group (range 2.0%–6.4%). Conversion to the open procedure was reported in 63 (4.6%) of the 1,380 cases initially randomized to LDG.

### 3.6. Main outcomes

#### 3.6.1. Intraoperative complications

**Figure 3:**
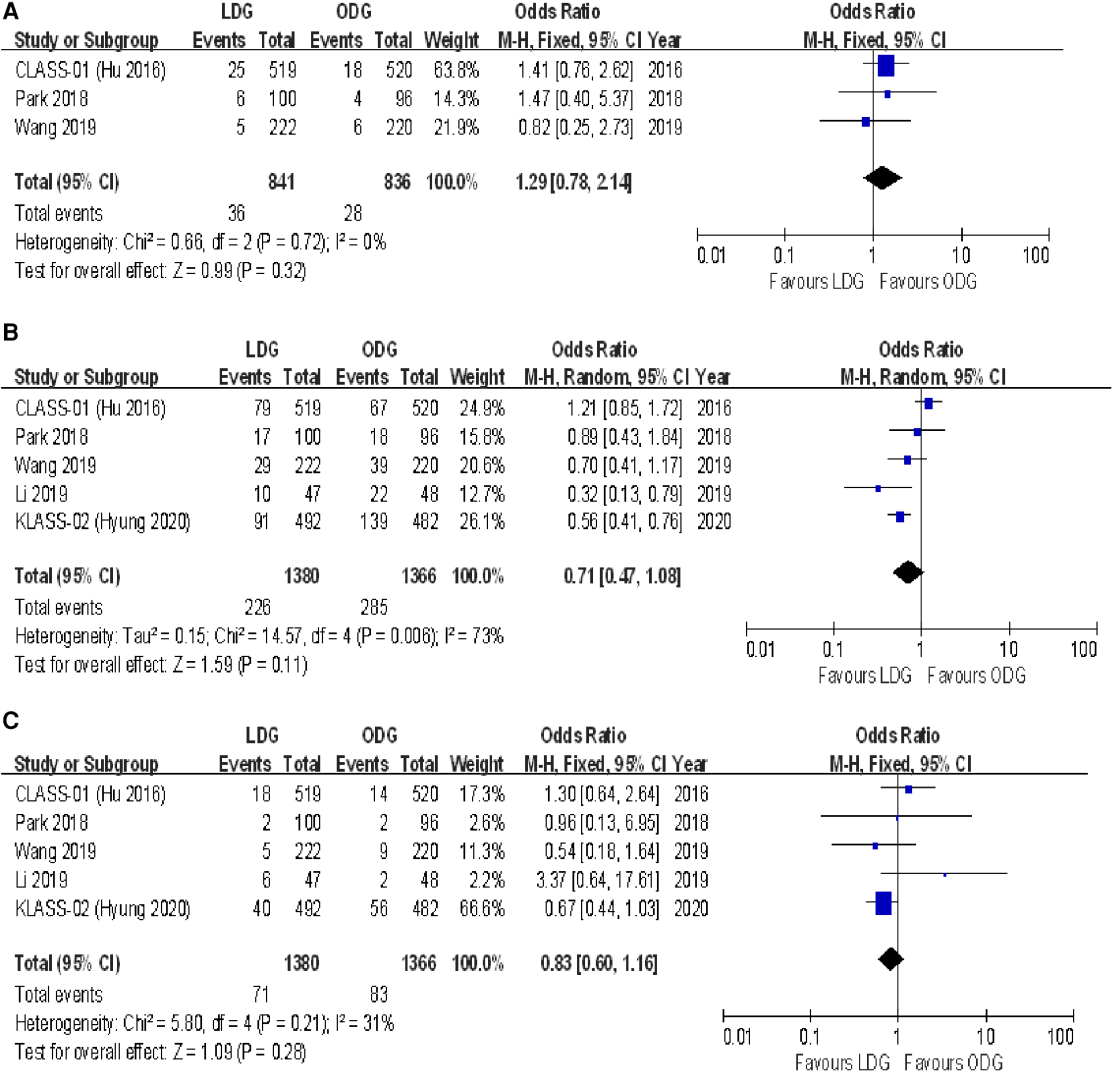
Forest plots of comparison between LDG and ODG on (**A**) intraoperative complications, (**B**) overall postoperative complications, and (**C**) serious postoperative complications. LDG, laparoscopic distal gastrectomy; ODG, open distal gastrectomy.

Three studies reported intraoperative complication rates. The rate was 4.3% (36/841) in the LDG group and 3.3% (28/836) in the ODG group. Heterogeneity was low (*I*^2 ^= 0%), so the fixed effects model was used. The pooled analysis of intraoperative complications revealed no significant differences between the LDG and the ODG groups [OR (CI): 1.29 (0.78–2.14)] (Figure 3A).

#### 3.6.2. Postoperative complications

Overall, postoperative complications within 30 days following surgery were reported in four of the included trials, and surgery-related complications occurring within the first 21 postoperative days were reported in the KLASS-02 trial (17). The postoperative complication rate was 16.4% (226/1,380) in the LDG group and 20.9% (285/1,366) in the ODG group. No significant differences were found between the two groups [OR (CI): 0.71 (0.47–1.08)], but there was high heterogeneity (*I*^2 ^= 73%) (Figure 3B).

The number of serious postoperative complications following surgery was reported in all included RCTs. These serious adverse events were assessed on the basis of the Accordion Severity Classification of Postoperative Complications (ASCPC) classification system in the trial reported by Park et al. (18), while serious postoperative complications of Clavien–Dindo grade 3 or higher were assessed in the remaining trials. The postoperative serious complication rate was 5.1% (71/1,380) in the LDG group and 6.1% (83/1,366) in the ODG group. A meta-analysis of these data revealed no differences between the two approaches [OR (CI): 0.83 (0.60–1.16)], and a low heterogeneity was found (*I*^2 ^= 31%) (Figure 3C).

#### 3.6.3. Oncologic outcomes

##### 3.6.3.1. Lymph nodes harvested

**Figure 4:**
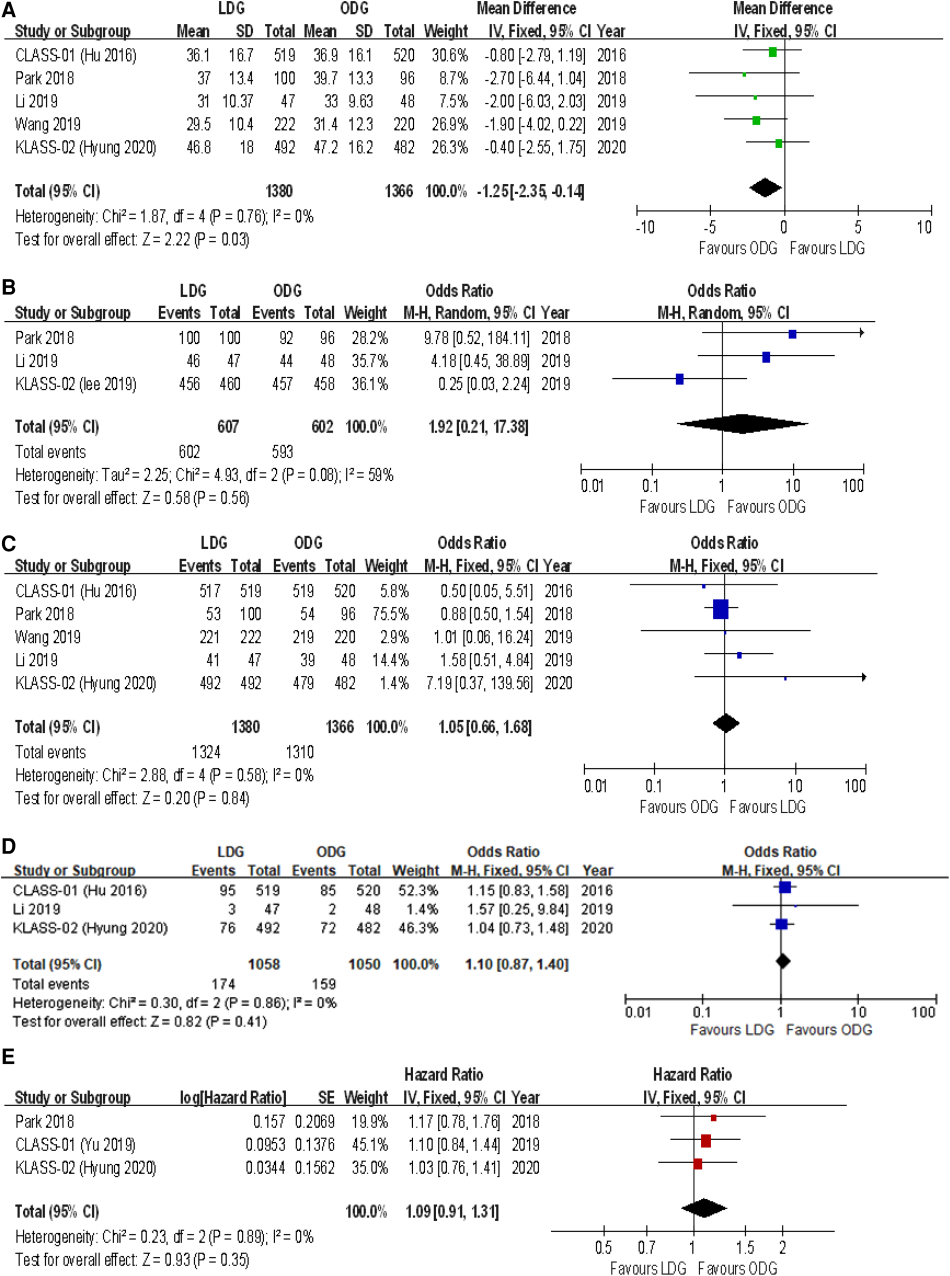
Forest plots of comparison between LDG and ODG on (**A**) harvested lymph nodes, (**B**) R0 resection, (**C**) compliance with D2 lymphadenectomy, (**D**) recurrence, and (**E**) 3-year disease-free survival. LDG, laparoscopic distal gastrectomy; ODG, open distal gastrectomy.

All trials reported the number of lymph nodes harvested. The lymph node retrieval was significantly higher in the ODG group by 1.3 nodes [WMD (CI): −1.25 (−2.35 to −0.14)] (Figure 4A).

##### 3.6.3.2. R0 resection

Three trials reported an R0 resection rate. There were no significant differences between the two groups [OR (CI): 1.92 (0.21–17.38)], but a high heterogeneity was found (*I*^2 ^= 59%) (Figure 4B).

##### 3.6.3.3. D2 lymphadenectomy

All trials reported the rates of D2 lymphadenectomy. There were no significant differences between the two groups [OR (CI): 1.05 (0.66–1.68)], and a low heterogeneity was reported (*I*^2 ^= 0%) (Figure 4C).

##### 3.6.3.4. Recurrence

Three trials reported recurrence rates at maximum follow-up. There were no significant differences between the two groups [OR (CI): 1.10 (0.87–1.40)], and a low heterogeneity was found (*I*^2 ^= 0%) (Figure 4D).

#### 3.6.4. Survival outcomes

Three-year disease-free survival (DFS) outcomes were reported in three studies. There were no significant differences between the two groups [HR (CI): 1.09 (0.91–1.31)], and there was low heterogeneity (*I*^2 ^= 0%) (Figure 4E).

### 3.7. Secondary outcomes

#### 3.7.1. Intraoperative outcomes

##### 3.7.1.1. Operative time

**Figure 5:**
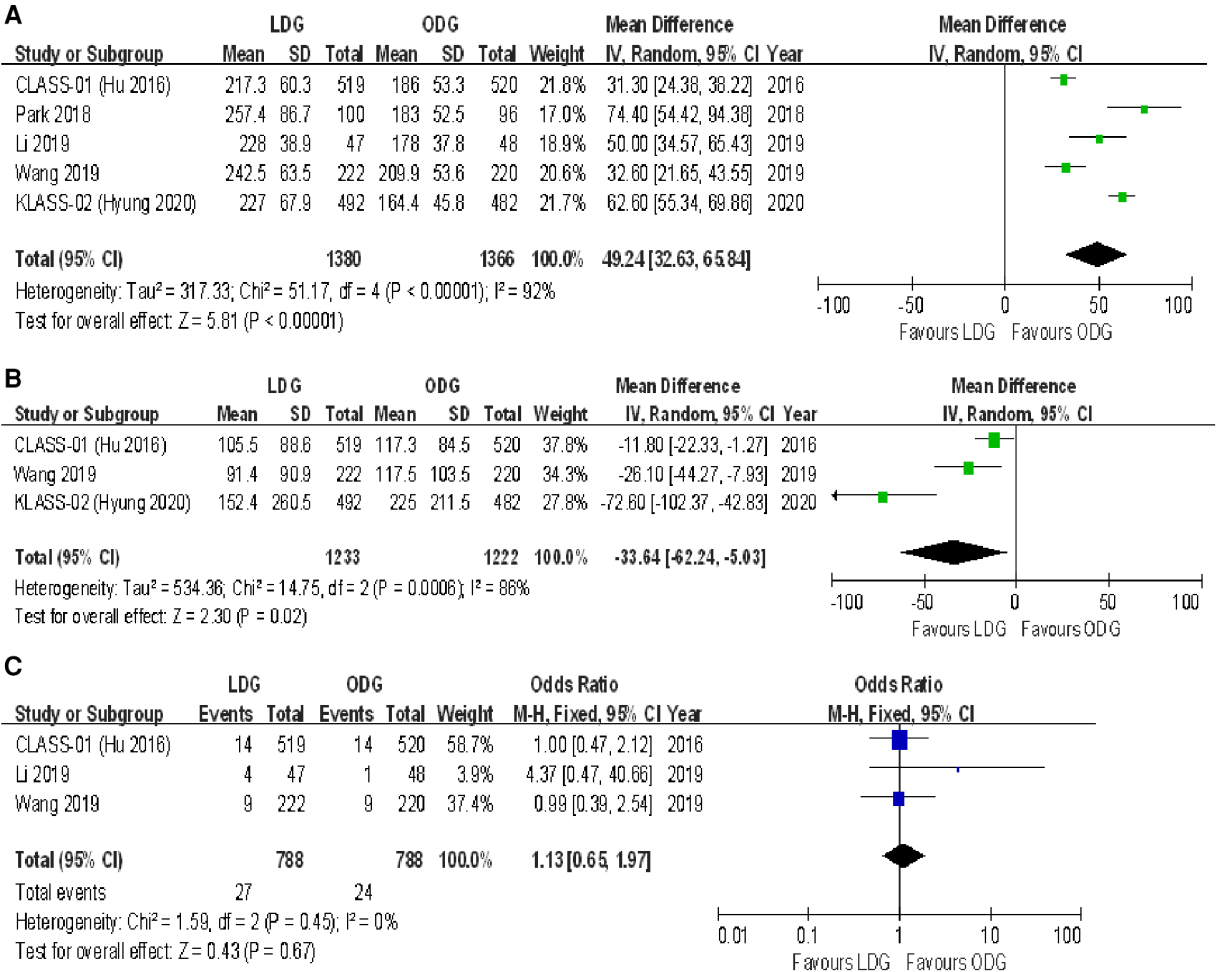
Forest plots of comparison between LDG and ODG on (**A**) operative time, (**B**) estimated blood loss, and (**C**) intraoperative blood transfusions. LDG, laparoscopic distal gastrectomy; ODG, open distal gastrectomy.

All trials reported operative times. There was a significantly longer operative time in the LDG group [WMD (CI): 49.24 (32.63–65.84)] with high heterogeneity (*I*^2 ^= 92%) (Figure 5A).

##### 3.7.1.2. Intraoperative blood loss

Three trials reported estimated blood loss during surgery. There was a significantly less intraoperative estimated blood loss in the LDG group [WMD (CI): −33.64 (−62.24 to −5.03)] with high heterogeneity (*I*^2 ^= 86%) (Figure 5B).

##### 3.7.1.3. Intraoperative blood transfusions

Three trials reported intraoperative blood transfusions. There were no significant differences between the two groups [OR (CI): 1.13 (0.65–1.97)], and a low heterogeneity was found (*I*^2 ^= 0%) (Figure 5C).

#### 3.7.2. Postoperative recovery

##### 3.7.2.1. Postoperative length of stay

**Figure 6:**
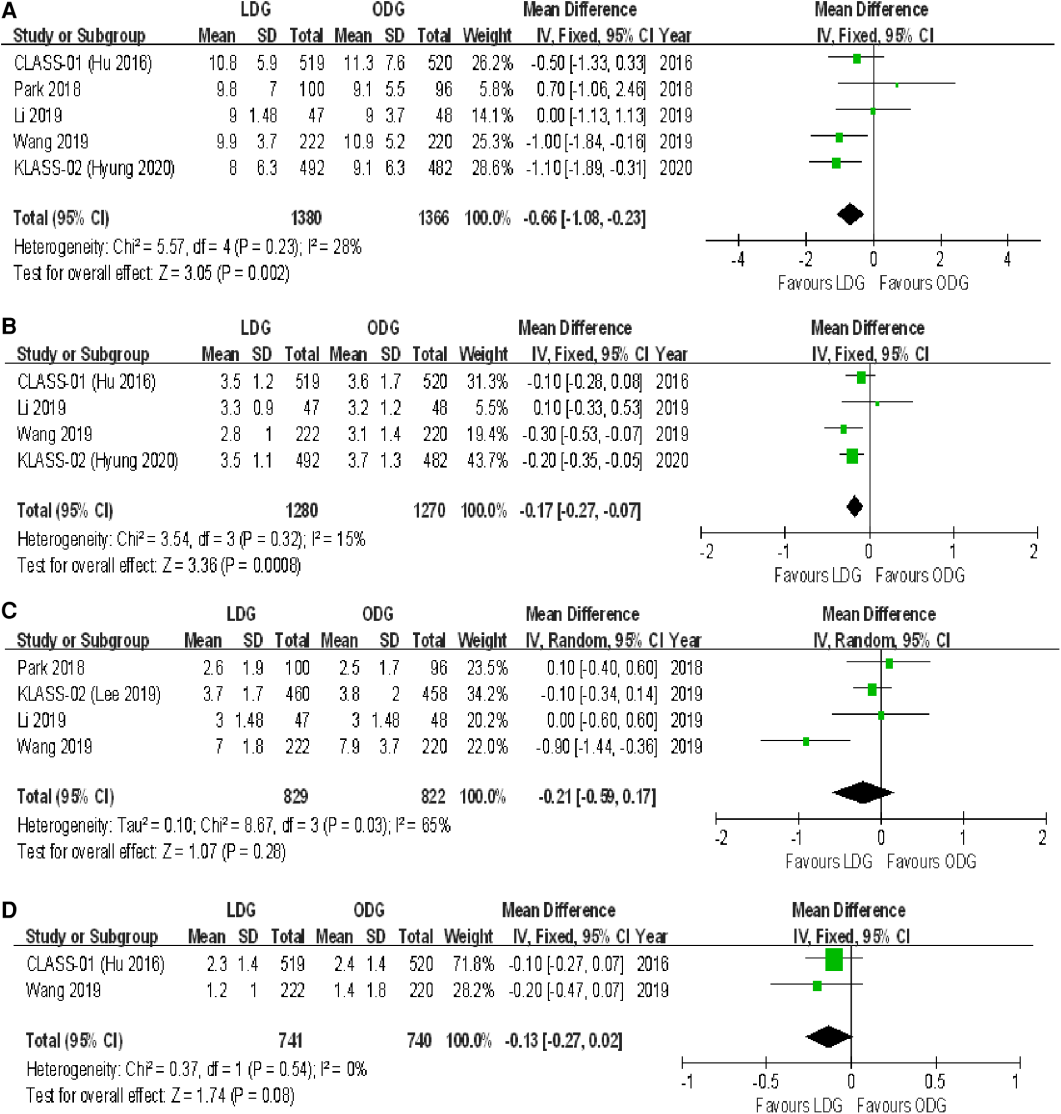
Forest plots of comparison between LDG and ODG on (**A**) postoperative length of stay, (**B**) time to first flatus, (**C**) time to first liquid diet, and (**D**) time to first ambulation. LDG, laparoscopic distal gastrectomy; ODG, open distal gastrectomy.

All trials reported postoperative length of stay. A significantly shorter length of stay was noted in the LDG group [WMD (CI): −0.66 (−1.08 to −0.23)] with low heterogeneity (*I*^2 ^= 28%) (Figure 6A).

##### 3.7.2.2. Time to first flatus

Four trials reported the time to first flatus. There was a significantly shorter time to first flatus in the LDG group [WMD (CI): −0.17 (−0.27 to −0.07)] with low heterogeneity (*I*^2 ^= 15%) (Figure 6B).

##### 3.7.2.3. Time to first liquid diet

Four trials reported the time to first liquid diet. No significant differences were found between the two groups [WMD (CI): −0.21 (−0.59 to 0.17)], but there was high heterogeneity (*I*^2 ^= 65%) (Figure 6C).

##### 3.7.2.4. Time to first ambulation

Two trials reported the time to first ambulation. No significant differences were found between the two groups [WMD (CI): −0.13 (−0.27 to 0.02)], and there was low heterogeneity (*I*^2 ^= 0%) (Figure 6D).

#### 3.7.3. Surgical margin

##### 3.7.3.1. Proximal margin

**Figure 7:**
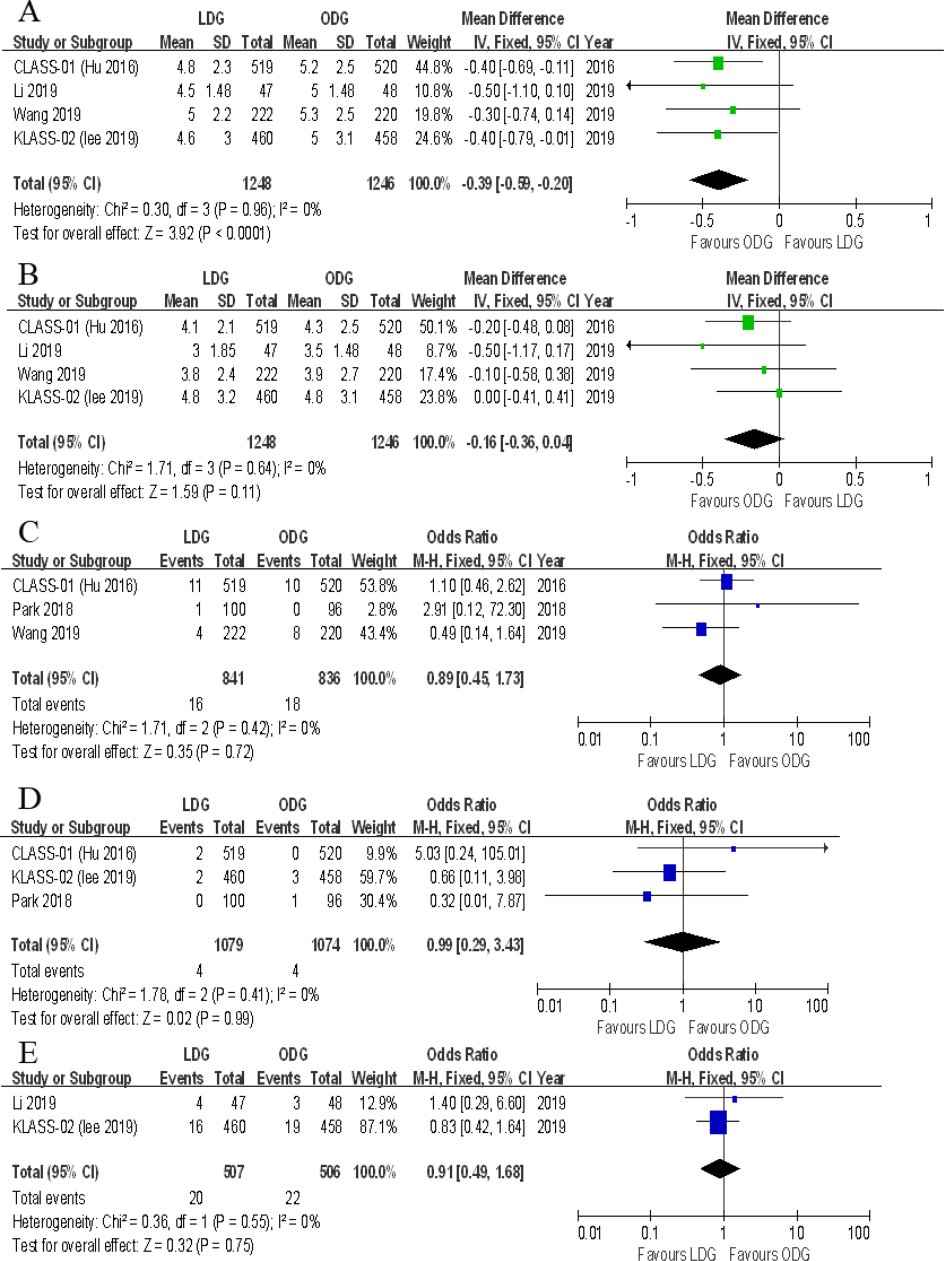
Forest plots of comparison between LDG and ODG on (**A**) proximal margin, (**B**) distal margin, (**C**) reoperation, (**D**) mortality, and (**E**) unplanned readmission. LDG, laparoscopic distal gastrectomy; ODG, open distal gastrectomy.

Four trials reported the proximal margin. There was a significantly less proximal margin in the LDG group [WMD (CI): −0.39 (−0.59 to −0.20)] with low heterogeneity (*I*^2 ^= 0%) (Figure 7A).

##### 3.7.3.2. Distal margin

Four trials reported the distal margin. There were no significant differences between the two groups [WMD (CI): −0.16 (−0.36 to 0.04)], and a low heterogeneity was noted (*I*^2 ^= 0%) (Figure 7B).

#### 3.7.4. Reoperation

Three trials reported the rates of reoperation. There were no significant differences between the two groups [OR (CI): 0.89 (−0.45 to 1.73)], and heterogeneity was low (*I*^2 ^= 0%) (Figure 7C).

#### 3.7.5. Mortality

All included RCTs reported postoperative mortality rates within 30 days. In two of the five RCTs, the mortality rate was 0% for both groups. A meta-analysis of the remaining three trials revealed no differences in short-term mortality rates between the two groups [OR (CI): 0.99 (0.29–3.43)], and a low heterogeneity was found (*I*^2 ^= 0%) (Figure 7D).

#### 3.7.6. Unplanned readmissions

Two trials reported unplanned readmissions. No significant differences were noted between the two groups [OR (CI): 0.91 (0.49–1.68)], and heterogeneity was low (*I*^2 ^= 0%) (Figure 7E).

#### 3.7.7. Type of postoperative complications

**Figure 8:**
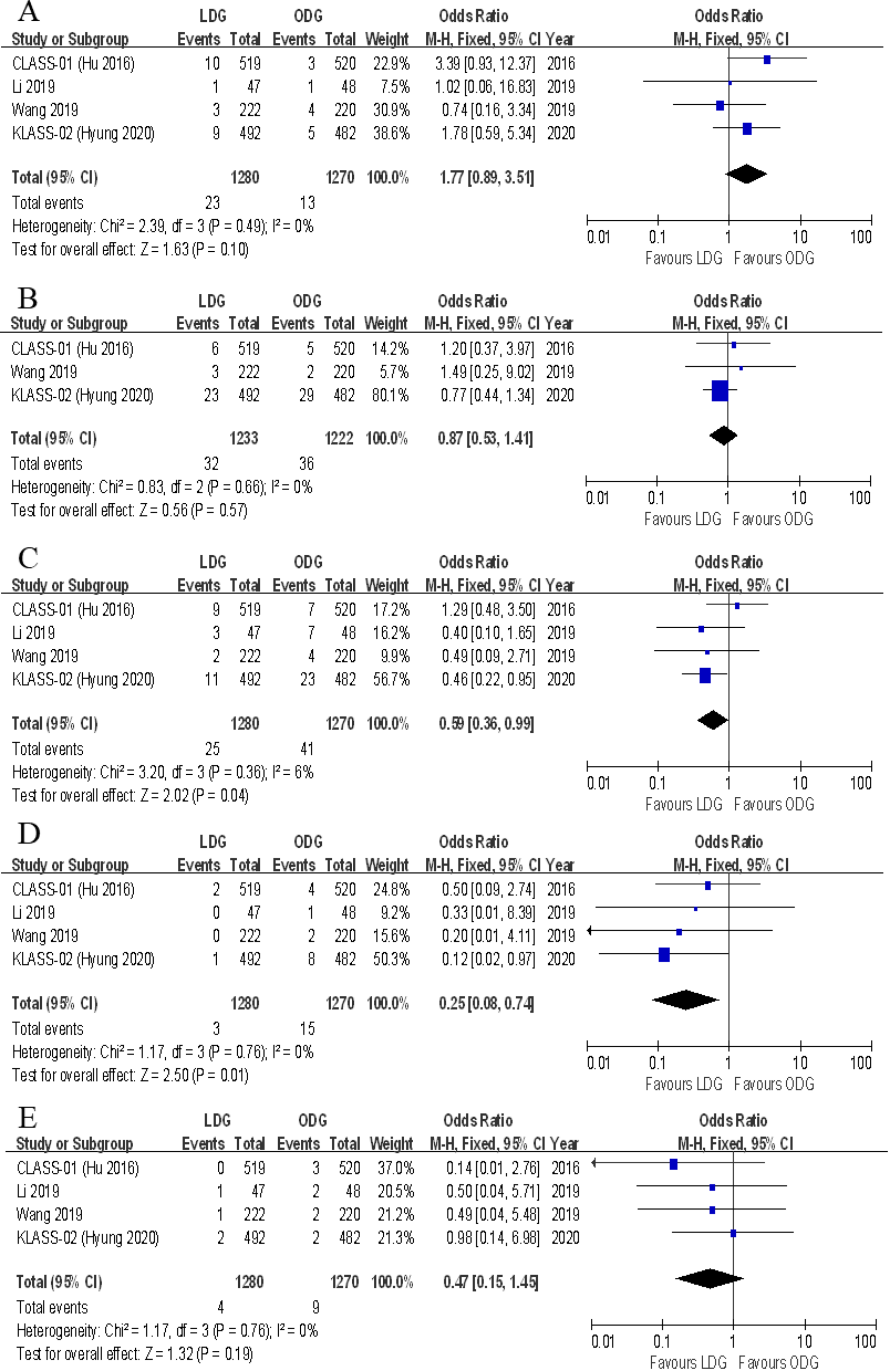
Forest plots of comparison between LDG and ODG on (**A**) anastomotic leakage, (**B**) wound infections, (**C**) intra-abdominal fluid collection, (**D**) intra-abdominal bleeding, and (**E**) intraluminal bleeding. LDG, laparoscopic distal gastrectomy; ODG, open distal gastrectomy.

**Figure 9:**
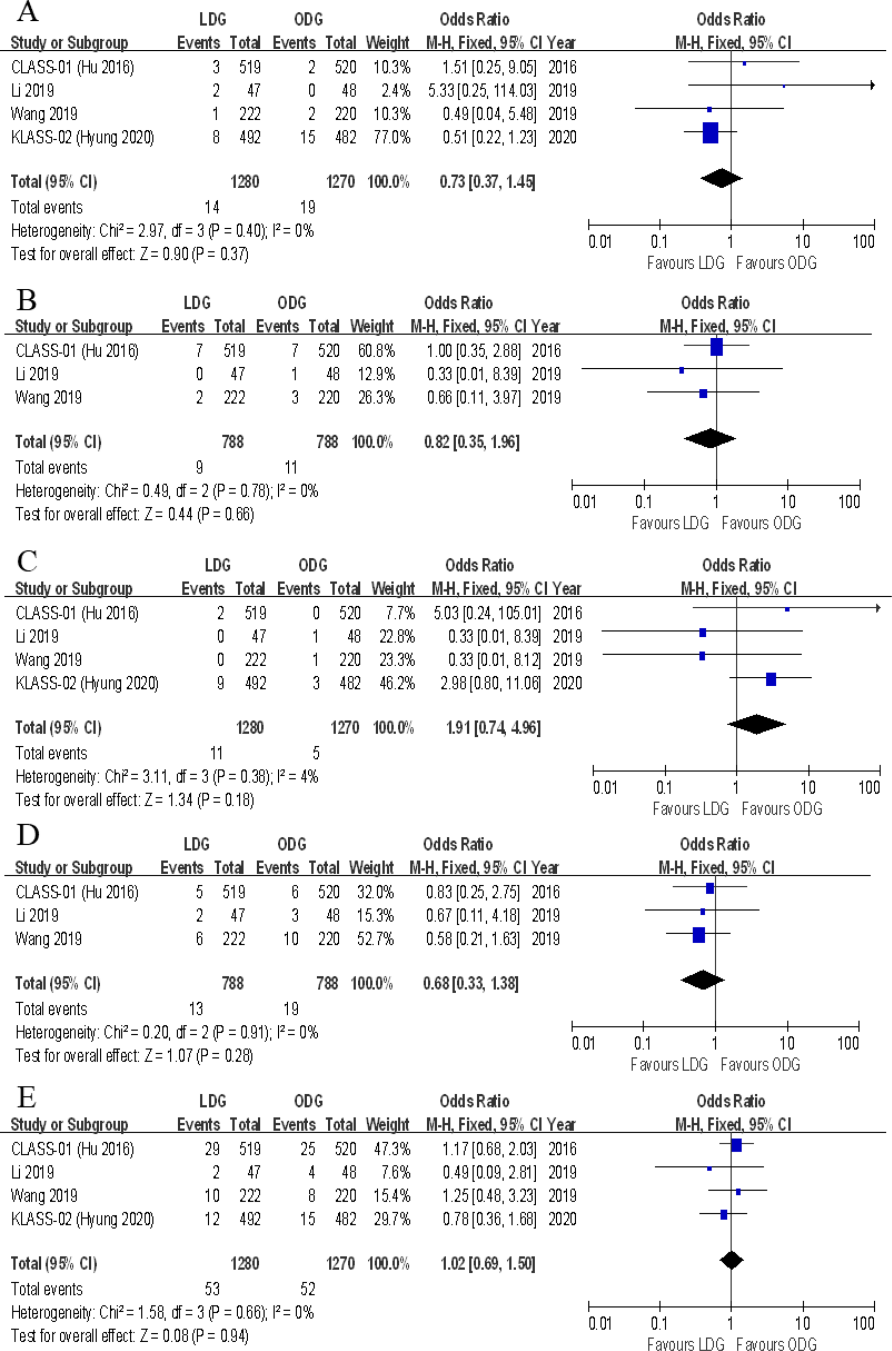
Forest plots of comparison between LDG and ODG on (**A**) ileus, (**B**) lymphatic leakage, (**C**) pancreatic fistula, (**D**) gastroplegia, and (**E**) pulmonary complications. LDG, laparoscopic distal gastrectomy; ODG, open distal gastrectomy.

Data on the types of postoperative complications were provided in four included trials. No significant differences were found between the two groups in terms of anastomotic leakage [OR (CI): 1.77 (0.89–3.51)], wound infections [OR (CI): 0.87 (0.53–1.41)], intraluminal bleeding [OR (CI): 0.47 (0.15–1.45)], ileus [OR (CI): 0.73 (0.37–1.45)], lymphatic leakage [OR (CI): 0.82 (0.35–1.96)], pancreatic fistula [OR (CI): 1.91 (0.74–4.96)], gastroplegia [OR (CI): 0.68 (0.33–1.38)], and pulmonary complications [OR (CI): 1.02 (0.69–1.50)] (Figures 8, 9). However, there were significantly less intra-abdominal fluid collection [OR (CI): 0.59 (0.36–0.99)] and bleeding [OR (CI): 0.25 (0.08–0.74)] in the LDG group (Figures 8C,D).

### 3.8. Sensitivity analysis, publication bias, and quality of evidence

**Figure 10:**
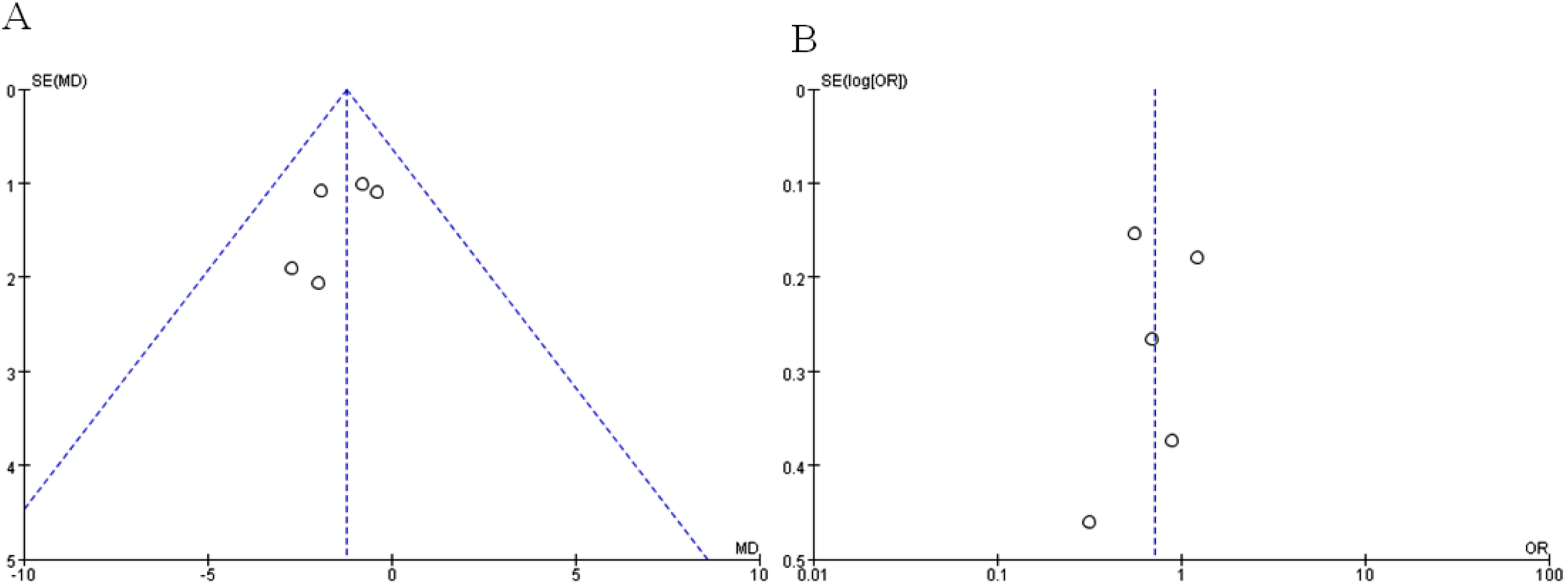
Funnel plots of (**A**) harvested lymph nodes and (**B**) overall postoperative complications for assessing publication bias.

A sequential exclusion of one study at a time was performed for the purposes of sensitivity analysis. The results of this analysis suggested that the pooled WMD value of harvested lymph nodes was significantly affected after excluding the Wang et al. RCT (WMD −1.01, CI −2.30 to 0.28, *p *= 0.13) or the Park et al. one (WMD −1.11, CI −2.26 to 0.05, *p *= 0.06), indicating that the overall effect size was volatile and therefore should be interpreted cautiously. A funnel plot analysis was conducted for examining the harvested lymph nodes and overall postoperative complications, which indicates that the publication bias of these studies is not obvious (Figure 10). The meta-analysis results with its certainty of evidence are partially summarized in Table 2. The overall quality of evidence ranged from moderate to very low.

**Table 2:** Summary of findings.

Outcomes	Anticipated absolute effects^a^ (95% CI)		Relative effect (95% CI)	No. of participants (studies)	Certainty of evidence (GRADE)
	Risk with ODG	Risk with LDG			
Intraoperative complications	33 per 1,000	43 per 1,000 (26–71)	OR 1.29 (0.78–2.14)	1,677 (3 RCTs)	⊕⊕◯◯ LOW^b,c^
Overall postoperative complications	209 per 1,000	148 per 1,000 (98–226)	OR 0.71 (0.47–1.08)	2,746 (5 RCTs)	⊕⊕◯◯ LOW^b,d^
Severe postoperative complications	61 per 1,000	51 per 1,000 (37–71)	OR 0.83 (0.60–1.16)	2,746 (5 RCTs)	⊕⊕⊕◯ MODERATE^b^
Harvested lymph nodes	The mean harvested lymph nodes were 39.71	The mean harvested lymph nodes in the LDG group were 1.25 lower (2.35 lower to 0.14 higher)	—	2,746 (5 RCTs)	⊕⊕⊕◯ MODERATE^b^
R0 resection	985 per 1,000	1,891 per 1,000 (207–17,119)	OR 1. 92 (0.21–17.38)	1,209 (3 RCTs)	⊕⊕◯◯ LOW^b,c,e^
D2 lymphadenectomy	986 per 1,000	1,942 per 1,000 (887–4,240)	OR 1. 97 (0.90–4.30)	2,746 (5 RCTs)	⊕⊕⊕◯ MODERATE^b^^,^^e^
Operative time	The mean operative time was 181.74 min	The mean operative time in the LDG group was 49.24 min higher (32.63 lower to 65.84 higher)	—	2,746 (5 RCTs)	⊕⊕◯◯ LOW^b,d^
Estimated blood loss	The mean estimated blood loss was 159.82 mL	The mean estimated blood loss in the LDG group was 33.64 mL lower (62.24 lower to 5.03 higher)	—	2,455 (3 RCTs)	⊕◯◯◯ VERY LOW^b,c,d^
Postoperative length of hospital stay	The mean postoperative length of hospital stay was 10.22 days	The mean postoperative length of hospital stay in the LDG group was 0.66 days lower (1.08 lower to 0.23 higher)	—	2,746 (5 RCTs)	⊕⊕⊕◯ MODERATE^b^
Time to first flatus	The mean time to first flatus was 3.54 days	The mean time to first flatus in the LDG group was 0.17 days lower (0.27 lower to 0.07 higher)	—	1,550 (4 RCTs)	⊕⊕⊕◯ MODERATE^b^

CI, confidence interval; LDG, laparoscopic distal gastrectomy; ODG, open distal gastrectomy; RCTs, randomized controlled trials; OR, odds ratio.

^a^ The risk in the intervention group (and its 95% confidence interval) is based on the assumed risk in the comparison group and the relative effect of the intervention (and its 95% CI).

^b^ The majority of studies did not blind participants, healthcare providers, and outcome assessors.

^c^ Low number of RCTs (n  = 3).

^d^ Strong evidence for statistical heterogeneity.

^e^ Wide confidence intervals.

## 4. Discussion

Several meta-analysis studies have compared laparoscopic gastrectomy with open gastrectomy for gastric cancer. However, most included articles are limited to patients suffering from EGC (2, 8). In addition, some included articles in previous meta-analyses were not restricted to distal gastric cancer and combined cases of patients who were exposed to the different levels of lymphadenectomy (8, 10). For AGC patients with the tumor located in the middle/lower part of the stomach indicating distal gastrectomy, it is not appropriate to directly provide treatment on the basis of the results reported in previous meta-analyses, which compared laparoscopic gastrectomy with open gastrectomy for AGC and reported data from different gastrectomy types, because they ignored the great amount of challenge involved in performing the procedure of total or proximal laparoscopic gastrectomy. Moreover, the anastomotic technique is particularly difficult to implement in total or proximal laparoscopic gastrectomy. Our meta-analysis compares laparoscopic gastrectomy with open gastrectomy for AGC patients in specific relation to distal gastrectomy. This meta-analysis summarizes the updated data of RCTs for AGC patients who specifically underwent distal gastrectomy and D2 lymphadenectomy, and it includes long-term follow-up RCTs published in the last few years.

Five RCTs available as full-text publications were included in the study and data were provided specifically with regard to AGC patients who underwent distal gastrectomy and D2 lymphadenectomy. These trials suggested steps to reduce intraoperative blood loss seen in the laparoscopic approach and to improve postoperative recovery rates. A recent meta-analysis of RCTs including mixed gastrectomy types for AGC found that short-term surgical outcomes and mortality and morbidity rates were not compromised by the laparoscopic approach as compared with the open gastrectomy one (10). Consistent with the results of this recent meta-analysis, our meta-analysis results also showed that the rates of intraoperative complications, overall morbidity, severe postoperative complications, intraoperative blood transfusion, reoperation, mortality, and unplanned readmission were similar between LDG and ODG for AGC patients. Similarly, the rates of R0 resection, D2 lymphadenectomy, distal margin, recurrence, and 3-year DFS were comparable in both groups. Of note, significant reductions in intraoperative blood loss, postoperative hospital stay, and time to first flatus were observed after the performance of LDG, and longer operation times were found for LDG.

Over the last two decades, great improvements in the quality of equipment and surgical techniques have widely influenced the management of AGC with a growing enthusiasm for laparoscopic procedures. Despite these advances, perioperative mortality rates were reported to be close to 1% for LDG in some studies. A study investigated 239 AGC patients from 10 Korean institutions and reported a mortality rate of 0.8% for LDG with lymph node dissection (22). The 30-day mortality rates of LDG and ODG for AGC patients in our meta-analysis were comparable (0.4% and 0.4%, respectively). With no statistically significant differences, intraoperative complication rates for LDG and ODG in our meta-analysis were 4.3% and 3.3%, respectively. In previous studies, the morbidity rates of LDG for AGC ranged from 8.0% to 24.2% (23, 24). Similarly, overall complication rates for LDG and ODG in our meta-analysis were 16.4% and 20.9%, respectively. The rates of serious postoperative complications have been previously reported to range from 2.1% to 6.0% for LDG (18, 25, 26). In the present analysis, LDG and ODG were associated with 5.1% and 6.1% serious postoperative complication rates, respectively. With regard to the issue of surgical safety, our meta-analysis showed no significant differences in terms of mortality, intraoperative complications, and overall and serious postoperative complications between the two groups.

Although the susceptibility to bias with regard to the surgeon's performance and experience level was generally discernible in these studies, a longer surgical time for LDG was a consistent finding across most studies (27). This could be attributed to a deprivation of surgeons' depth perception, decreased flexibility, and increased difficulty of dissection of lymph nodes and total omentectomy in laparoscopic procedures (28–30). In addition, the constant need to change instruments and clean cameras also increased the surgical time of LDG (31). Our analysis showed reduced intraoperative blood loss in LDG, but no significant increase of intraoperative blood transfusion was found in ODG. Although the difference in blood loss was only 33.6 mL in our study and high heterogeneity was found among the included studies, these suggest a consistent difference. In other procedures such as colectomy and rectectomy, reduced intraoperative blood loss in the laparoscopic group was a consistent finding when compared with laparoscopic and open techniques (8, 32). This could be attributed to the fact that advanced laparoscopic surgical instruments facilitated a meticulous hemostasis and prevented unexpected bleeding (33). In addition, time to first flatus and postoperative hospital stay were found to be significantly reduced in LDG compared with ODG. However, the question whether this reduced hospitalization can counterbalance the higher costs of laparoscopic procedures for AGC patients remains unanswered.

With regard to the type of postoperative complications, most major surgical complications (anastomotic leakage, wound infections, ileus, lymphatic leakage, and pancreatic fistula) of gastric surgery were comparable between the two groups (9, 10). In the present analysis, LDG and ODG were associated with 1.8% and 1.0% anastomotic leakage rates, respectively. LDG appeared to have a potential higher risk of anastomotic leakage compared with ODG because of the increased difficulty of performing intracorporeal anastomosis by using laparoscopic tools or performing extracorporeal anastomosis by using minilaparotomy in conditions of restricted space and vision (16). In the present meta-analysis, LDG for AGC patients provided the benefits of lower intra-abdominal fluid collection and bleeding, which could be attributed to the performance of more delicate maneuvers through visual magnification during the procedure (17). Notably, although no statistically significant differences were found in most studies, more instances of anastomotic leakage occurred after the performance of LDG, whereas probably less instances of intra-abdominal fluid collection and bleeding occurred post-LDG, suggesting that these differences should be considered when performing laparoscopic surgery.

The most striking finding in our meta-analysis was of decreased lymph node retrieval in the LDG versus ODG. Previous studies noted that LDG might attenuate lymph node dissection, which may limit the application of LDG for AGC patients (34). Subsequently, a series of RCTs and nonrandomized comparative studies reported similar efficacy of lymph node clearance between LDG and ODG groups, but a small reduction of harvested lymph nodes in the LDG group was a consistent finding across these studies. In this meta-analysis, lymph node yield was found to be significantly reduced in the LDG group, although D2 lymphadenectomy was comparable between the groups. Although this held true for our meta-analysis, the biggest two RCTs in our study found no statistical difference in the number of harvested lymph nodes between LDG and ODG (16, 17). The sensitivity analysis suggested that the overall effect size was volatile and further studies were needed to examine this aspect. The volatile pooled result of the harvested lymph nodes in our meta-analysis may be attributed to a small number of RCTs restricted to distal gastric cancer and the inclusion of a relatively small sample. An adequate lymph node clearance is essential for the purposes of cancer staging and is important for reducing recurrence and improving overall survival (OS) rates (35–37). The results of this meta-analysis showed that lymph node yield significantly reduced by 1.3 nodes in the LDG group, but the mean number of the harvested lymph nodes in this group exceeded 29 lymph nodes among all included studies. Although a lower proximal margin was obtained after the performance of LDG, this did not impact oncologic adequacy because the R0 dissection rates were similar between the two groups. Although the operation settings of surgical space and vision were different in laparoscopic and open surgeries, the generally high motivation levels of surgeons could have resulted in the reduced yield of lymph nodes and proximal margin of LDG. Surgeons might adopt a more conservative and discreet approach while performing lymphadenectomy and a proximal cut edge during laparoscopic surgery because both laparoscopic lymphadenectomy at a deep lymph node station and intracorporeal/extracorporeal anastomosis fashioning can be challenging, whereas there are larger spaces for lymphadenectomy and gastroenterostomy under direct vision during the open procedure. As the surgical margins fulfilled R0 resection criteria and the dissected lymph nodes were sufficient, we opine that the open or laparoscopic procedure did not determine the survival time of gastric cancer (38).

The long-term survival outcome was important for the application of LDG in AGC patients. Currently, three RCT studies (CLASS-01, KLASS-02, and COACT1001) have reported long-term survival outcomes concerning LDG and ODG for AGC (12, 13, 18). The results including the rates of OS, DFS, and recurrence could be used to evaluate the long-term oncologic outcomes of surgical procedures (39). However, data on 5-year OS and DFS are limited. Thus, 5-year OS was reported only in CLASS-01 and was 72.6% for the LDG group and 76.3% for the ODG group, with no significant differences (*p* = 0.19) (12). Correspondingly, data on 3-year OS in CLASS-01 was 83.1% for the LDG group and 85.2% for the ODG group (*p* = 0.28) (11). Within KLASS-02, 3-year OS was 90.6% for the LDG group and 90.3% for the ODG group (*p* = 0.96) (13). Consistent with these results, the pooled analysis of 3-year DFS in our meta-analysis also showed no significant differences between LDG and ODG. For patients with AGC, tumor recurrence was found in some patients in the 4th or 5th postoperative year. A recurrence rate of up to 20% has been previously reported (40). In the present analysis, the recurrence rate at maximum follow-up was 16.4% for LDG and 15.1% for ODG, with no significant differences. These results indicate that LDG is a feasible surgical procedure for treating AGC in terms of long-term survival outcomes.

Surgeons’ experience and quality control have a major impact on surgical outcomes. The number of cases needed to overcome the learning curve of LDG has been to show inconclusive results. Studies that evaluated the learning curve of LDG for AGC have shown a significant reduction in operation time, blood loss, postoperative hospital stay, morbidity, and an increase of lymph node yield after the performance of 40 LDG procedures with D2 lymphadenectomy (41). In the present meta-analysis, all included studies reported a previous experience of surgeons performing an LDG and highlighted the surgeons' credentials for a proficient performance of an LDG. In addition, the measures of surgical quality control were described in all RCTs through intraoperative images, videos, and checklist evaluation to ensure the adequacy of lymphadenectomy and gastric resection. These data extracted from RCTs conducted in high-volume tertiary centers may not be applicable to small grassroots hospitals and therefore should be interpreted carefully. As D2 lymphadenectomy is a technically demanding procedure, we suggest that LDG for AGC patients should be performed by experienced surgeons who completed the learning curve in high-volume tertiary hospitals.

There were several limitations in this study. First, all included RCTs were carried out in high-volume tertiary hospitals of East Asia (three in China and two in Korea), which could be a potential source of bias limiting the generalizability of these findings. Second, approximately 25% of included subjects preoperatively diagnosed to have AGC were downstaged to EGC after the release of the postoperative pathological results. Third, only one RCT in the current meta-analysis reported outcomes in patients who underwent preoperative neoadjuvant chemotherapy, and further research studies are needed to comprehensively evaluate outcomes after providing neoadjuvant treatment. Fourth, even though our analyses included only RCTs, the quality of evidence remained in the moderate to very low range, which could be partly attributed to the non-blinding of patients and surgeons, the small sample size, the different laparoscopic skills of the surgeons, and the varying methods of anastomosis and quality control. Last but not the least, there lies the possibility that Clavien–Dindo classification system for assessing postoperative complications used in included trials will fail to consider many such complications in the analysis, such as blood transfusion, the use of parenteral nutrition, antibiotic treatment of urinary tract infections, and pneumonia or fistulas treated conservatively. In addition, most included trials studied complications only up to a period of 30 postoperative days in gastrectomies, which also gives rise to the possibility of leaving out of the analysis complications suffered by many patients. Therefore, it is important to use the standard outcome definition and classify postoperative complications and record 90-day morbidity rates in future studies (42).

This study summarizes the highest quality data comparing LDG with ODG for AGC specific to distal gastric cancer. Data from five RCTs suggest that LDG with D2 lymphadenectomy for AGC has similar short-term surgical and long-term survival outcomes to ODG when performed by experienced surgeons in high-volume hospitals. LDG may result in a reduced retrieval of lymph nodes and lower proximal margin, and at the same time, LDG does not impair oncologic adequacy as D2 lymphadenectomy and R0 resection rates are comparable between the two groups. In addition, recent studies have confirmed the feasibility of the surgical safety of LDG for AGC but may not have sufficient power to assess the differences in postoperative complications between LDG and ODG. Further research studies are necessary to investigate whether LDG has any advantages over ODG for the management of AGC.

## Data Availability

The original contributions presented in the study are included in the article/Supplementary Material, further inquiries can be directed to the corresponding author.
