# Supplementary material for: Laparoscopic vs. open distal gastrectomy for locally advanced gastric cancer: A systematic review and meta-analysis of randomized controlled trials
Source: Front Surg. 2023 Feb 17;10:1127854. doi: 10.3389/fsurg.2023.1127854 (PMC9982133; doi:10.3389/fsurg.2023.1127854)
Supplement: Supplementary file 1 [file Table1.docx]

**Laparoscopic Versus Open Distal Gastrectomy for Locally Advanced Gastric Cancer: A Systematic Review and Meta-analysis of Randomized Controlled Trials**

**Supplementary Table 1.** Patient recruitment and surgical details of the trials included in the meta-analysis

| Study | Trial ID | Recruitment period | Inclusion criteria | Exclusion criteria | Surgical details | Outcome |
| --- | --- | --- | --- | --- | --- | --- |
| CLASS-01 | NCT01609309 | 2012.9 - 2014.12 | Age: 18-75 years Tumor: primary gastric adenocarcinoma confirmed pathologically by endoscopic biopsy cT2-4aN0-3M0 at preoperative evaluation according to AJCC Cancer Staging Manual, 7th Edition Other: expected curative resection via distal subtotal gastrectomy with D2 lymphadenectomy | Previous upper abdominal surgery (except laparoscopic cholecystectomy). Previous gastrectomy, endoscopic mucosal resection, or endoscopic submucosal dissection. Enlarged or bulky regional lymph node diameter larger than 3 cm based on preoperative imaging Previous neoadjuvant chemotherapy or radiotherapy | The extent of gastrectomy and D2 lymph node dissection was based on the Japanese gastric cancer treatment guidelines. The type of reconstruction was determined by the surgeon’s experience and preference. Extracorporeal anastomosis using a minilaparotomy was recommended during laparoscopic surgery | Primary outcome: 3-year DFS rate Secondary outcomes: morbidity and mortality rates within 30 days, 3-year overall survival rate, 3-year recurrence pattern, postoperative recovery course, inflammatory and immune response, intra- and post-operative complication |
| KLASS-02 | NCT01456598 | 2011.11 - 2015.4 | Age: 20-80 years Tumor: histologically confirmed gastric adenocarcinoma; cT2 to cT4a and cN0 to cN1 in preoperative gastroscopy, endoscopic ultrasound, and/or abdominal computed tomography Other: tumor can be resected by distal gastrectomy in curative intention | Possible distant metastasis, existence of other malignancies within last 5 years, history of previous gastric resection, gastric cancer-related complications (complete obstruction or perforation), or history of gastric cancer treatment by endoscopic resection, chemotherapy and/or radiotherapy | Standard distal gastrectomy with D2 lymph node dissection including total omentectomy. Reconstruction was selected among gastroduodenostomy (Billroth I), loop gastrojejunostomy (Billroth II), or Roux-en-Y gastrojejunostomy depending on tumor location and/or surgeon’s preference. Hand sewing or stapling method and extracorporeal or intracorporeal method for anastomosis could be selected according to surgeon’s preference | Primary outcome: 3-year DFS rate Secondary outcomes: postoperative morbidity and mortality, postoperative recovery, quality of life, 3-year overall survival rate |
| Wang 2019 | NCT02464215 | 2014.3 - 2017.8 | Age: ≥18 years Tumor: pathologically confirmed primary gastric adenocarcinoma by endoscopic biopsy; preoperative cancer stage cT2-4aN0-3M0 (according to AJCC-7th TNM staging) Other: tumor located in the lower part of the stomach, potentially resectable by subtotal gastrectomy and D2 lymph node dissection | Surgical history of upper abdomen (except laparoscopic cholecystectomy); Previous gastrectomy, including endoscopic submucosal dissection and endoscopic mucosal resection; Integrated or enlarged lymph node with maximum diameter larger than 3 cm according to preoperative imaging; Other malignant diseases (within 5 years); Preoperative chemotherapy, immunotherapy or radiotherapy; Complications (bleeding, perforation, or obstruction) required emergency surgery due to primary gastric malignancy | Gastrectomy was performed with total omentectomy. The extent of lymphadenectomy adhered principles of Japanese gastric cancer treatment guidelines. Reconstruction was not limited in this trial. Surgeons performed standard Billroth-I (B-I), Billroth-II (B-II), or Roux-en-Y fashion according to their preferences | Operating time, estimated blood loss, open conversion, intraoperative blood transfusion, resection margins, number of lymph nodes harvested, length of incision, intraoperative complications, postoperative recovery course, postoperative hospital stay, postoperative morbidity and mortality |
| Park 2018 | NCT01088204 | 2010.6 - 2011.10 | Age: 20-80 years Tumor: histologically confirmed gastric adenocarcinoma; cT2 to cT4a and cN0 to cN3 in preoperative gastroscopy, and abdominal computed tomography Other: Neoadjuvant chemotherapy was not administered to the enrolled patients | Participation in another trial, language problems, lack of compliance, mental inability, synchronous or previous malignant disease, systemic administration of corticosteroids, unstable angina or myocardial infarction within 6 months of the trial, severe respiratory disease, American Society of Anesthesiologists score >3, previous major abdominal surgery, previous chemo- or radiotherapy, inadequate liver, kidney, and bone-marrow functions, and Eastern Cooperative Oncology Group status >1 | The surgical procedures are performed according to the guidelines of the Japanese Research Society for Gastric Cancer. Extension of lymph node dissection may be applied according to surgeon’s estimation in case of further suspicious involvement. The duodenum is dissected distal to the pyloric ring and the stomach should be dissected proximally with a margin of at least 3 cm. Distal margin should be at least 1 cm. Reconstruction is also left to the surgeon’s discretion (Billroth I, Billroth II, Roux-en-Y) | Primary outcome: noncompliance rate of lymph node dissection Secondary outcomes: surgical, oncologic outcomes, and survival rate |
| Li 2019 | NCT02404753 | 2015.4 - 2017.11 | Age: 18-80 years Tumor: histologically confirmed gastric adenocarcinoma with clinical stage of cT2-4aN+M0 by preoperative evaluation Other: Three weeks after the last cycle of neoadjuvant chemotherapy patients with resectable tumors were randomized | Previous upper abdominal surgery (except laparoscopic cholecystectomy) Previous gastric surgery (including diagnosis procedure such as endoscopic submucosal dissection and endoscopic mucosal resection) Other malignant diseases in 5 years Medium or severe renal damage | A standard distal gastrectomy with D2 lymphadenectomy in accordance with the Japanese Gastric Cancer Treatment Guidelines.  In both groups, the reconstruction method was selected from the standard procedures of Billroth I/II or Roux-en-Y, depending on the surgeon’s discretion and preference | Primary outcome: 3-year DFS rate Secondary outcomes: morbidity and mortality rates within 30 days, surgical radicality, postoperative recovery course, adjuvant  chemotherapy completion status |

LDG, laparoscopic distal gastrectomy; ODG, open distal gastrectomy; DFS, disease-free survival.

**Supplementary Table 2.** Surgeons' qualification and control measures for surgical quality within the trials included in the meta-analysis

| Study | Number of surgeons or institutions | Surgeons’ qualification | Quality control |
| --- | --- | --- | --- |
| CLASS-01 | 15 surgeons at 14 institutions | Have performed at least 50 distal gastrectomies with D2 lymphadenectomy using open and laparoscopic approaches, have performed at least 300 gastrectomies for patients with AGC annually at each institute, and were determined to be qualified surgeons by the CLASS academic committee on the basis of the evaluation of unedited videos of both their open and laparoscopic gastrectomy with D2 lymphadenectomy procedures | Surgical quality control was maintained by using mandatory intraoperative photographs that identified specific surgical fields, the resection margin of the specimen, and the abdominal incision. Five photos were required to verify the surgical quality of the D2 lymph node clearance as follows: (1) The area between the pancreatic tail and the lower pole of the spleen, (2) The pancreatic head and infrapyloric area, (3) The right side of the suprapancreatic area, (4) The left side of the suprapancreatic area, (5) The lesser curvature area. These photos were reviewed, and feedback on the assessment was regularly provided to the investigators |
| KLASS-02 | Multicenter | Surgeons were obliged to have performed ≥50 cases of each LDG and ODG. More than 80 cases were asked to be performed in the surgeon’s institute each year. Surgeons were validated through a separate clinical trial (KLASS-02-QC, NCT01283893), in which surgeons submitted their unedited videos of 3 laparoscopic and 3 open distal gastrectomies with D2 lymphadenectomy and each video was reviewed by 5 domestic or international experts according to the KLASS-02 evaluation sheet which was developed by KLASS-02 steering committee. | Unedited video in laparoscopic surgery or photos of surgical fields in open surgery for every 10 cases were collected and reviewed. The regular steering committee reviewed the trend of morbidity of each surgeon and, if needed, provided a technical feedback to the surgeon with high morbidity. |
| Wang 2019 | 9 surgeons in 5 centers | Have already conducted at least 60 ODG and 60 LDG with D2 lymphadenectomy previously. Each participating institute could perform at least 80 gastrectomies for advanced gastric cancer patients each year. | Intraoperative photographs and unedited videos were mandatory required and monitored by the study chair to control the surgical quality. Ten photos were uploaded for each participant. Among them, five pictures were taken for lymph node dissection fields, four for the lesion and resection margins of specimens, and one for the abdominal incision. |
| Park 2018 | Multicenter | Had performed at least 30 LDG procedures before the start of this study | To standardize the open and laparoscopic D2 lymphadenectomy procedures, all surgeons attended 10 video seminars to observe unedited videos of the surgical procedure before the start of this trial. To evaluate the D2 lymphadenectomies, a list of checkpoints created to determine their success. |
| Li 2019 | 1 surgeon in a single center | Has performed more than 600 laparoscopic gastrectomy procedures in total and has an annual surgical volume of approximately 300 (including both laparoscopic and open gastrectomy) | The unedited full operation videos of patients undergoing laparoscopic surgery and the photographs of lymph node dissection fields, surgical incisions, and specimen of patients undergoing open surgery will be preserved. The specific requirements for the photographs are as follows: Lymph node dissection surgical field; Area under the pylorus; The disconnection part of the left gastroepiploic vessels; The right side of the upper edge of the pancreas; The left side of the upper edge of the pancreas; The right side of the cardia and small curvature of the residual stomach; After the skin incision is closed; Postoperative specimens |

LDG, laparoscopic distal gastrectomy; ODG, open distal gastrectomy; AGC, advance gastric cancer.
